# Treatment of human pre-B acute lymphoblastic leukemia with the Aurora kinase inhibitor PHA-739358 (Danusertib)

**DOI:** 10.1186/1476-4598-11-42

**Published:** 2012-06-21

**Authors:** Fei Fei, Min Lim, Sabine Schmidhuber, Jürgen Moll, John Groffen, Nora Heisterkamp

**Affiliations:** 1 Section of Molecular Carcinogenesis, Division of Hematology/Oncology and The Saban Research Institute of Children’s Hospital Los Angeles, Los Angeles, CA 90027, USA; 2 Current address: Laura Bassi Centre of Expertise, Theraped/Forschungsprogramm für Rezeptorbiochemie und Tumorstoffwechsel, Universitätsklinik für Kinder- und Jugendheilkunde, Paracelsus Medizinische Privatuniversität, Vienna, Austria; 3 Department of Cell Biology, Nerviano Medical Sciences, Nerviano, MI, I-20014, Italy; 4 Leukemia Research Program, Children’s Hospital Los Angeles, Los Angeles, USA; 5 Leukemia and Lymphoma Program, Norris Comprehensive Cancer Center, University of Southern California, Los Angeles, CA USA; 6 Departments of Pediatrics and Pathology, Keck School of Medicine, University of Southern California, Los Angeles, CA 90033, USA; 7 Division of Hematology/Oncology, Ms#54, Children’s Hospital Los Angeles, 4650 Sunset Boulevard, Los Angeles, CA 90027, USA

**Keywords:** Ph-positive, Aurora kinase inhibition, Drug resistance, Stromal support, Co-culture, Farnesyltransferase inhibitor, Lonafarnib, Dasatinib, p53, Combination drug treatment

## Abstract

**Background:**

Treatment of Philadelphia chromosome-positive acute lymphoblastic leukemias (Ph-positive ALL) with clinically approved inhibitors of the Bcr/Abl tyrosine kinase frequently results in the emergence of a leukemic clone carrying the T315I mutation in Bcr/Abl, which confers resistance to these drugs. PHA-739358, an Aurora kinase inhibitor, was reported to inhibit the Bcr/Abl T315I mutant in CML cells but no preclinical studies have examined this in detail in human ALL.

**Results:**

We compared the sensitivity of human Bcr/Abl T315I, Bcr/Abl wild type and non-Bcr/Abl ALL cells to this drug. PHA-739358 inhibited proliferation and induced apoptosis independently of Bcr/Abl, the T315I mutation, or presence of the tumor suppressor p53, but the degree of effectiveness varied between different ALL samples. Since short-term treatment with a single dose of drug only transiently inhibited proliferation, we tested combination treatments of PHA-739358 with the farnesyltransferase inhibitor Lonafarnib, with vincristine and with dasatinib. All combinations reduced viability and cell numbers compared to treatment with a single drug. Clonogenic assays showed that 25 nM PHA-739358 significantly reduced the colony growth potential of Ph-positive ALL cells, and combined treatment with a second drug abrogated colony growth in this assay. PHA-739358 further effectively blocked Bcr/Abl tyrosine kinase activity and Aurora kinase B *in vivo,* and mice transplanted with human Bcr/Abl T315I ALL cells treated with a 3x 7-day cycle of PHA-739358 as mono-treatment had significantly longer survival.

**Conclusions:**

PHA-739358 represents an alternative drug for the treatment of both Ph-positive and negative ALL, although combined treatment with a second drug may be needed to eradicate the leukemic cells.

## Introduction

Although significant advances have been made in the treatment of acute lymphoblastic leukemia (ALL) especially in children, only 30-40% of adults have a long-term survival [1]. A major subclass of ALL with a specially poor prognosis in both adults and children is that of Philadelphia chromosome-positive ALL [2,3]. The Ph chromosome is generated by a reciprocal t(9;22) translocation. It is found in around 30% of cases of adult ALL and is the hallmark of chronic myeloid leukemia (CML). The deregulated tyrosine kinase activity of the chimeric Bcr/Abl protein in these leukemias phosphorylates a broad range of substrates, many of which are key cellular signal transduction proteins [4-6]. The tyrosine kinase inhibitor imatinib became the first-line therapy in the conventional treatment of CML, with a relatively selective targeting of the ATP binding site of Bcr/Abl [2,7-10]. However, the emergence of resistance to imatinib remains a major problem especially for those patients with advanced CML (with progression in about 7% of patients after 7 years), or with Ph-positive ALL. This is due to point mutations in the Bcr/Abl kinase domain, including the most frequent T315I and E225K mutations [4,6,7,11]. Second-generation tyrosine kinase inhibitors, such as nilotinib, dasatinib and bosutinib are capable of targeting the majority of imatinib-resistant mutations, but none of them are effective against leukemia cells harboring the T315I mutation [11-14]. Thus, the need to find a more effective treatment for leukemia patients with this mutation is obvious.

Aurora kinases are key regulators of cell division [15,16] and deregulation of this activity can result in aneuploidy and carcinogenesis [17]. Therefore, they are attractive targets for anticancer therapy [18,19]. Several small molecule inhibitors of Aurora kinases with various properties are in clinical trials including PHA-739358 (Danusertib) [20,21], MLN8054 [22] and AZD1152 [23]. PHA-739358 is a pan-Aurora kinases inhibitor with activity against all Aurora kinase family members (A, B and C) [24,25]. Interestingly, and of importance for the potential use of this compound against poor-prognosis ALL, Gontarewicz *et al*, using Bcr/Abl constructs transfected into the BaF3 cell line, showed that PHA-739358 is also effective against imatinib-resistant Bcr/Abl mutants including the T315I [24]. A determination of the crystal structure of the T315I Abl kinase domain in complex with PHA-739358 showed that the drug interacts with the active conformation of Abl kinase [26]. Currently, preliminary evidence for anti-tumor activity of PHA-739358 has been seen in various advanced refractory cancers, and phase II studies in solid tumors are ongoing [20].

In this report, we performed preclinical studies in the presence of stroma *in vitro* as well as *in vivo,* to explore the application of PHA-739358 for treatment of a variety of primary human acute lymphoblastic leukemia cells including those belonging to the Ph-positive ALL subclass and harboring the T315I mutation. We conclude that PHA-739358 could be considered for the treatment of patients with different subtypes of ALL in combination with other drugs to potentiate its cytostatic and cytotoxic effects.

## Results

### PHA-739358 reduces viability of acute lymphoblastic leukemia cells including those with the Bcr/Abl T315I mutation

To determine the impact of the Bcr/Abl status on the efficacy of PHA-739358, we treated human ALL cells including BLQ1, Pt2 (Bcr/Abl^+^, T315I mutation), UCSF02, TXL2 (Bcr/Abl^+^, wild type), US7, US7R (non-Bcr/Abl) and mouse 8093 and Bin2 cells (Bcr/Abl^+^, wild type) with increasing concentrations of PHA-739358 for 72 hours. In Phase I-II clinical trials, a C_max_ of 4–6 μM/h was observed for CML patients harboring the T315I mutation when PHA-739358 was administered at 330 mg/m^2^/day [27]. Therefore, we used clinically relevant and achievable concentrations of up to 5 μM PHA-739358 in our experiments.

**Figure 1:**
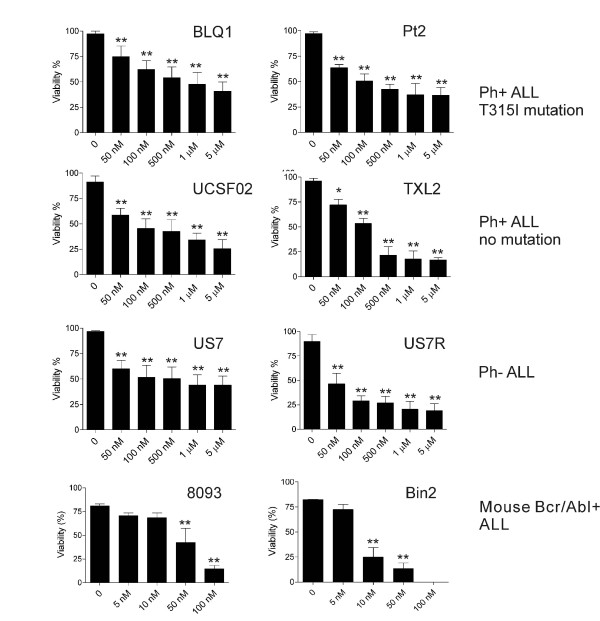
**PHA-739358 decreases viability of human and mouse lymphoblastic leukemia cells. **Human acute lymphoblastic leukemia cells including BLQ1, Pt2 (Bcr/Abl^+^, T315I mutation), UCSF02, TXL2 (Bcr/Abl^+^, wild type), US7, US7R (non-Bcr/Abl) and mouse Bcr/Abl^+^ 8093 and Bin2 leukemia cells were cultured in the presence of stroma. Viable cell counts were determined by Trypan blue exclusion assay after treatment for 72 hours with the indicated amounts of PHA-739358. Error bars indicate SD. Results shown are one of two independently performed experiments in triplicate with similar results. (*p < 0.05, **p <0.001).

As shown in Figure 1, increasing concentrations of PHA-739358 caused a cytotoxic effect on all the leukemia cells tested as measured by the decreased viability of the cultures. There was no correlation between the type of ALL and sensitivity to the drug. Compared to human leukemia cells, mouse 8093 and Bin2 cells were significantly more sensitive to PHA-739358. Although these murine Bcr/Abl ALL cells contain an identical transgene, they also exhibited different sensitivity to this drug.

### PHA-739358 induces apoptosis and leads to an accumulation of cells with ≥ 4N DNA content

**Figure 2:**
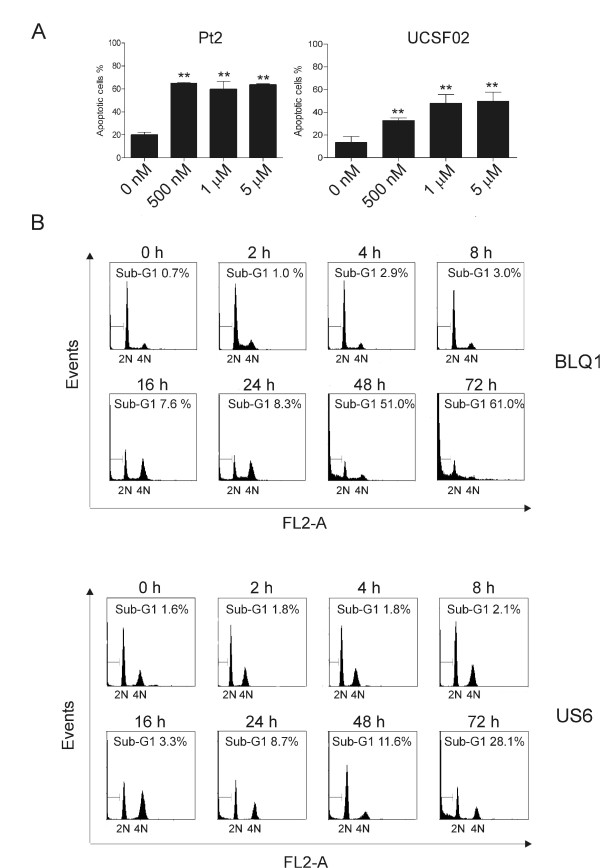
**PHA-739358 induces apoptosis and cell cycle arrest. (A)**. Pt2 or UCSF02 cells were treated with increasing concentrations of PHA-739358 for 48 hours in triplicate. Apoptotic cells were identified by flow cytometry as Annexin V/PI double-positive. **(B)**. BLQ1 Ph-positive or Ph-negative US6 cells were exposed to 1 μM PHA-739358 for 48 hours and cell cycle distribution was assessed by flow cytometry at different time points as indicated. (*p < 0.05, **p <0.001).

The ability of PHA-739358 to induce apoptosis was measured by Annexin V/PI staining in Pt2 and UCSF02 cells treated with increasing concentrations of the drug for 48 hours. As demonstrated in Figure 2A, PHA-739358 induced apoptosis both in Pt2 and UCSF02 cells. Since inhibition of Aurora kinases causes endoreduplication and polyploidy [28], we assessed DNA content at different time points in Ph-positive BLQ1 and Ph-negative US6 cells treated with PHA-739358. Mutations and deletions of p53 are rare in ALL and of the samples examined here, only US6 had defective p53 function (by p53 deletion; not shown). In agreement with previous findings using Aurora kinase inhibitors (PHA-739358, VX-680, AZD1152) in other types of cancer cells [24,28-30], PHA-739358 caused accumulation of BLQ1 and US6 cells with more than or equal to 4 N DNA content as early as 16 hours (Figure 2B). Moreover, 1 μM PHA-739358 generated polyploid (4 N DNA) cells and produced a significant reduction in viability, as assessed by the percentage of cells in the sub-G1 DNA content.

### PHA-739358 targets both Bcr/Abl and Aurora kinase activities

**Figure 3:**
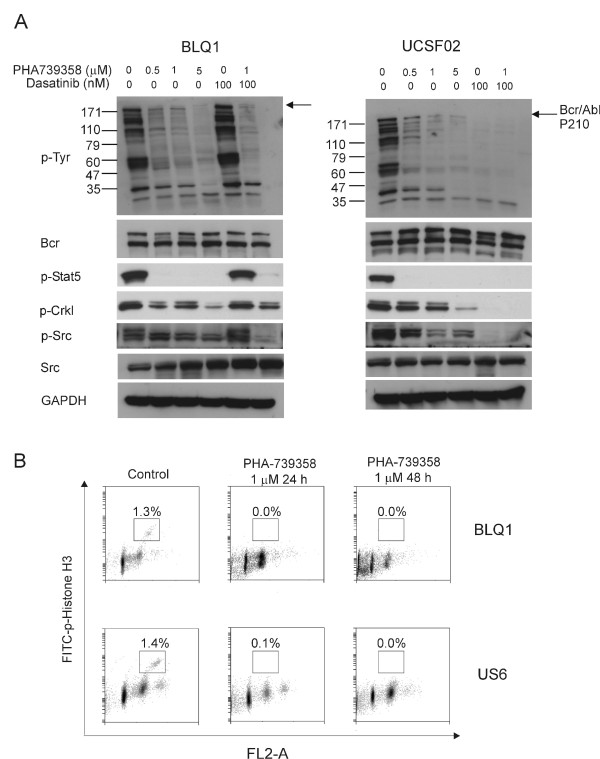
**PHA-739358 eliminates activity of both Bcr/Abl and Aurora kinase. (A)**. BLQ1 (Ph-positive T315I mutation) and UCSF02 (Ph-positive no mutation) cells were incubated with the indicated concentrations of PHA-739358 with or without dasatinib (100 nM) for 24 hours. Phosphorylation status of Bcr/Abl, Stat5, Crkl and Src was assessed by Western blot. Blots were stripped and reprobed with Bcr, Src and Gapdh as loading controls. **(B)**. BLQ1 and US6 cells were exposed to 1 μM PHA-739358 for 24 and 48 hours and the percentage of phospho-histone H3 (Ser10) positive cells was defined by flow cytometry.

PHA-739358 was reported to inhibit both Bcr/Abl kinase and Aurora kinase *in vitro*[31], whereas dasatinib targets Bcr/Abl and Src-family kinases [32]. To examine this in human Ph-positive ALL cells, the effect of PHA-739358 on the activity of Bcr/Abl was determined by examining the phosphorylation of overall tyrosine, of Crkl and of Stat5. A concentration of 1 μM PHA-739358 blocked the generation of total phosphotyrosine significantly in both T315I Bcr/Abl BLQ1 and wild type Bcr/Abl UCSF02 cells (Figure 3A). As shown in Figure 3A, increasing concentrations of PHA-739358 decreased the phosphorylation status of Crkl. Stat5 phosphorylation was completely inhibited even at 1 μM PHA-739358. Treatment with 100 nM dasatinib (concentration chosen based on the maximum plasma concentration range [33]) also induced a distinct inhibition in phosphotyosine, p-Crkl, p-Stat5 and p-Src in UCSF02 cells. However, as expected, there was no effect of dasatinib in BLQ1 cells harboring the T315I mutation. Similar results were also obtained with cell cycle analysis (data not shown). We also evaluated the effect of PHA-739358 on Aurora B kinase activity, by measuring inhibition of phosphorylation of its substrate histone H3 at position Ser10 [34] using Ph-positive BLQ1 and Ph-negative US6 cells. As shown in Figure 3B, 24 hours of treatment with 1 μM PHA-739358 caused an obvious reduction of p-histone H3 to 0.1% compared to 1.6% and 1.4% in untreated BLQ1 and US6 cells respectively.

### ALL cells resume proliferation after short-term PHA-739358 treatment

**Figure 4:**
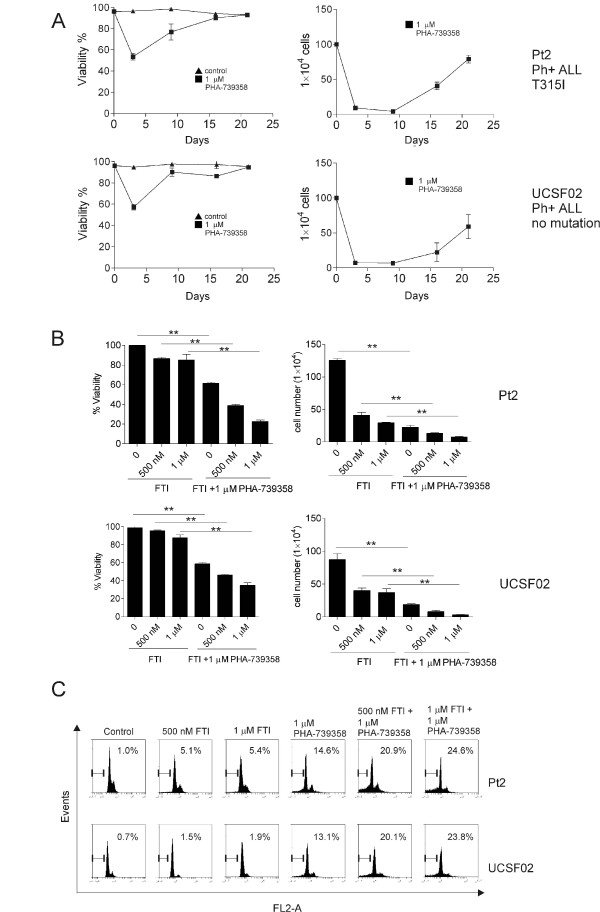
**Combination treatment with PHA-739358 in Ph-positive ALL cells. (A)**. Pt2 or UCSF02 cells were exposed to 1 μM PHA-739358 for 3 days. After 3 days, the drug was removed and the cells were cultured without PHA-739358. Viability (left panels) and cell numbers (right panels) were measured. **(B)**. Pt2 and UCSF02 cells were treated with the FTI Lonafarnib and PHA-739358 individually or together for 3 days. Cell viability and viable cell numbers were determined by Trypan blue exclusion assay. **(C)**. Cell cycle analysis of Pt2 and UCSF02 treated with FTI alone or combined with PHA-739358 for 48 hours. (*p < 0.05, **p <0.001).

As mentioned above (Figure 1), in the presence of stroma, 1 μM PHA-739358 treatment for 3 days left 50% of the Pt2 and UCSF02 cells in an apparently viable state. In the study by Gontarewicz et al. [24], they observed that PHA-739358 significantly inhibited the proliferation of K562 cells *in vivo* during 10 days of treatment. However, when the application of the drug was terminated, K562 cells started to proliferate again. We therefore examined the effect of short-term treatment of PHA-739358, followed by no treatment. Pt2 and UCSF02 cells were exposed to 1 μM of PHA-739358 for 3 days in the presence of stroma, after which drug was removed. As shown in Figure 4A (left panels), after removal of PHA-739358 on day 3, viability of both Pt2 and UCSF02 cultures increased gradually. By day 16, cells began to proliferate again (Figure 4A, right panels) and the viability of the cells reached a level similar to that of the control culture. However, such cells remained sensitive to re-treatment with PHA-739358, and Bcr/Abl exhibited a sensitivity similar to that displayed by the orignal non-drug treated cells (data not shown). This indicates that the ALL cells had not acquired genetic resistance to this Aurora kinase inhibitor.

### Combination treatment significantly increases effect of PHA-739358

To investigate the possibility of increasing the effect of PHA-739358 on cell cycle inhibition, we tested it in combination with a second drug that also affects cell cycle. Farnesyltransferase inhibitors (FTI) inhibit farnesylation of mitotic proteins CENP-E and CENP-F while Aurora kinases inhibitors will inhibit the phosphorylation of CENP-E [35]. We therefore treated Pt2 (T315I mutation Bcr/Abl) and UCSF02 (wild-type Bcr/Abl) with 500 nM or 1 μM of the FTI Lonafarnib (SCH66336) alone or together with 1 μM PHA-739358 for 3 days. As shown in Figure 4B (left panels), exposure of Pt2 or UCSF02 to 500 nM or 1 μM FTI alone resulted in minimal toxicity as judged by viability, but consistent with its inhibition of cell cycle, did prevent cell proliferation (right panels). Interestingly, combined treatment with PHA-739358 and the FTI resulted in a substantial increase in cell death in both Pt2 and UCSF02 cells. We also assessed DNA content by treating Pt2 and UCSF02 cells with FTI with or without PHA-739358 for 48 hours. Notably, co-administration of PHA-739358 with FTI resulted in a striking increase in the sub-G1 compartment (Figure 4C).

To determine the ability of PHA-739358 to augment the efficacy of drugs currently in use in a clinical setting for therapy of Ph + ALL, we treated Pt2 cells (T315I mutation Bcr/Abl) with 2.5 nM or 5.0 nM vincristine alone or together with 1 μM PHA-739358 for 3 days. As demonstrated in Additional file 1: Figure S1A, exposure of Pt2 to 2.5 nM or 5.0 nM vincristine alone decreased cell viability to 80 and 50%, respectively. The combined treatment with PHA-739358 and vincristine further significantly reduced cell viability and cell numbers. A combination of dasatinib with PHA-739358 in wild type Bcr/Abl UCSF02 had a similar effect (Additional file 1: Figure S1B).

The growth inhibitory effect of PHA-739358 on human ALL cells was further confirmed using a colony formation assay. As shown in Additional file 2: Figure S2, 10 nM PHA-739358 led to about 55% and 25% reduction of colony numbers in Pt2 and UCSF02 cells, respectively, compared with the controls. PHA-739358 at a concentration of 25 nM almost completely inhibited the colony formation of both Pt2 and UCSF02 cells. Combined treatment of PHA-739358 with FTI, vincristine or dasatinib completely inhibited the growth of Pt2 and UCSF02 as assessed by colony formation assay. Therefore, we confirmed that a significant portion of the effect of PHA-739358 on human ALL cells was due to its growth inhibitory effect.

### *In vivo* efficacy of PHA-739358 on Bcr/Abl cells with T315I mutation

**Figure 5:**
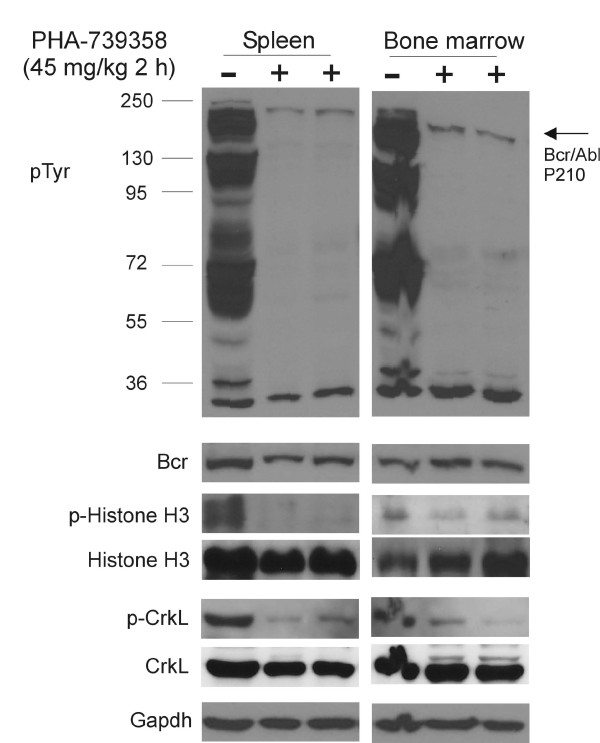
**Monitoring of PHA-739358 treatment effect *in vivo *on molecular targets. **Fully leukemic NSG mice transplanted with Pt2 Ph-positive ALL were treated with PHA-739358 (45 mg/kg, iv). Samples of one non-treated (−) and two drug-treated (+) mice are shown. Two hours after injection, spleen and bone marrow were collected. The expression of p-Tyr, p-histone H3 (Ser 10), p-Crkl, histone H3 and Crkl was assessed by Western blot. Bcr and Gapdh serve as a loading control.

To examine the efficacy of PHA-739358 *in vivo*, Pt2 cells with the T315I mutation were transplanted into NSG mice via tail vein injection. After mice developed leukemia, we evaluated the inhibitory effects of PHA-739358 on the phosphorylation levels of tyrosine, histone H3 and Crkl 2 hours after drug administration. As shown in Figure 5, there was a significant down-regulation of the levels of total phosphotyrosine, of phospho-Crkl and of phospho-histone H3 by Western blot, both in bone marrow and spleen of mice transplanted with leukemia cells, indicating that it was able to inhibit both Bcr/Abl and Aurora B activities *in vivo*.

**Figure 6:**
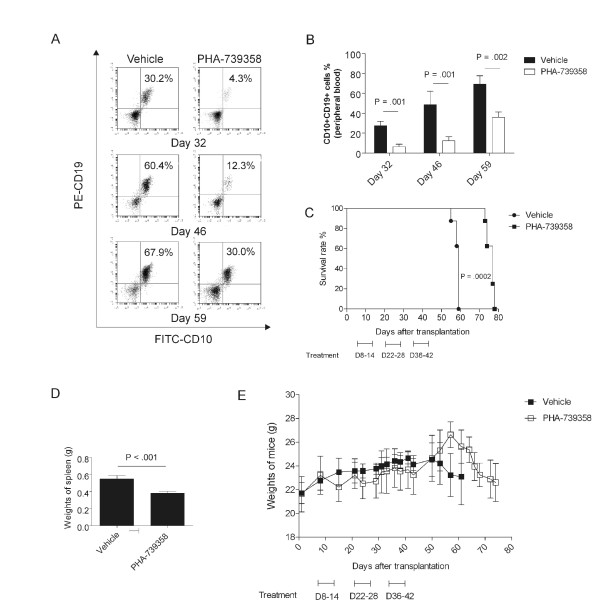
***In vivo *efficacy of PHA-739358 on human leukemia cells. **Pt2 cells (2x10^6^) were transplanted in NSG mice, which were started on treatment on day 7 with three cycles of PHA-739358. **(A)**. Representative example of flow cytometry analysis of the human ALL cells (CD10^+^CD19^+^) in peripheral blood at different time points. **(B)**. Bar graph representing the percentage of CD10^+^CD19^+^ cells in peripheral blood (n = 8). **(C)**. Survival of mice in PHA-739358 group (n = 8, 30 mg/kg, iv, qd, 7 days on/7 days off x 3 cycles) was significantly prolonged compared to the vehicle treatment group (n = 8) (p = 0.0002). **(D)**. Weights of spleens of vehicle treatment group compared to those of PHA-739358 treatment group (p < 0.001). **(E)**. Weights loss or gain of mice during treatment.

We also measured the effect of PHA-739358 on the outcome of leukemia. Seven days after transplantation of Pt2 ALL cells into NSG mice, we administered three cycles of 30 mg/kg PHA-739358 treatment. One cycle consisted of daily injections for 7 days, followed by a 7-day break. We monitored the percentage of leukemia cells in the peripheral blood by flow cytometry. Figure 6A, B shows that, in comparison with vehicle-treated mice, PHA-739358 treated mice showed significantly decreased amounts of leukemia cells in the peripheral blood on day 32 (30% *vs* 5%), day 46 (45% *vs* 10%) and day 59 (70% *vs* 35%) after transplantation. However, peripheral blood still contained around 5% of leukemia cells even after two cycles of PHA-739358 treatment at day 32. When the administration of PHA-739358 was terminated on day 42, leukemia cells started to proliferate again in the treatment group. Figure 6B demonstrates that from day 46 to day 59, the percentage of leukemia cells in the PHA-739358 treated group increased from about 10% to 40%, compared to the control group in which an increase from 55% to 70% was measured.

Consistent with the percentage of leukemia cells observed in peripheral blood, the mice in the control group died rapidly, with a median survival time (MST) of 59 days, while the mice in the PHA-739358 treated group showed a distinctly prolonged survival time (MST = 77)(Figure 6C). Interestingly, splenomegaly of mice was less pronounced in the PHA-739358 treated group than in the vehicle treated group (p < 0.001, Figure 6D). Treatment with PHA-739358 appeared to be well-tolerated, since there were no significant differences in weight loss or gain (Figure 6E) or changes in physical appearance between the two groups.

## Discussion

The current study tested the use of PHA-739358 for the treatment of Ph-positive ALL *in vitro* and *in vivo.* Since PHA-739358 has dual activity against both Bcr/Abl and Aurora kinases, one could expect that the inhibition of Ph-positive ALL would be more profound than that of Ph-negative ALL. However, we could not detect an increased effect on the Ph-positive samples, and Ph-positive samples with or without the T315I mutation did not differ significantly in sensitivity. Our results with the mutants agree with Gontarewicz et al., who reported that PHA-739358 was effective against imatinib-resistant Bcr/Abl mutants including those with the T315I mutation in human and mouse leukemia cell lines as well as in CD34^+^ cells from an imatinib-resistant CML patient [24].

We did notice that for some samples, dose escalation did not result in a proportionally larger response. This effect was quite marked in, for example, Pt2. Although treatment with 500 nM PHA-739358 caused a drop in viability to around 40% in 3 days, a 10-fold increased dose of 5 μM did not increase the percentage of apoptotic cells or decrease the viability. Similarly, a 100-fold difference (50 nM – 5 μM) of drug exposure of UCSF02 did not cause a corresponding increased loss in viability. The lack of dose-proportionality might be due to saturation of the mechanism at low concentrations. Indeed, data from the colony formation assays show that a significant part of the effects of PHA-739358 are due to its growth inhibitory activity, which is seen at a concentration as low as 10 nM.

In other cancers, deletion or mutation of p53 has been shown to result in resistance to the induction of apoptosis. We therefore examined whether any of the ALL samples contained p53 mutations using RT/PCR but none were detected. Only US6 showed lack of an RT/PCR product, suggesting bi-allelic loss of p53 (results not shown). These cells reacted to the drug by accumulation of cells with a DNA content of >4N but the amount of cells with a sub-G1 DNA content was less than BLQ1, which is wild type for p53. Interestingly, in hepatocellular carcinoma cell lines, Benten *et al* also found that PHA-739358 exhibits activity against both p53-wild type and mutated cancers [36].

In initial studies using 8093 murine Bcr/Abl transgenic ALL cells transplanted into C57Bl recipients, we found that, compared to control mice, mice that had been treated with 30 mg/kg/bid i.v. PHA-739358 for 5 days survived significantly longer than controls (MST 25 days; control MST 17 days; results not shown). However, mice relapsed shortly after termination of the treatment. The behavior of the leukemia cells *in vivo* was modeled, to some extent, by *in vitro* co-culture with stroma. In that system, a 3-day treatment with PHA-739358 caused a significant reduction in cell numbers of Pt2 and UCSF02 and suppressed cell proliferation for 6 days or more, but, consistent with Gontarewicz *et al*[24] cells subsequently resumed proliferation with restored Bcr/Abl activity.

Because of this, we examined the effect of treatment with PHA-739358 in combination with a second drug. Since the primary mechanism of action of PHA-739358 is to inhibit the cell cycle, we combined it with a farnesyltransferase inhibitor, which has a similar molecular target Farnesyltransferase inhibitors were originally developed to prevent Ras oncoprotein prenylation. However, FTIs also inhibit the farnesylation of mitotic proteins CENP-E and CENP-F, which mediate chromosomal capture and alignment [37,38], while Aurora kinases phosphorylate CENP-E [35]. FTIs were in phase II/III clinical trials for treatment of a variety of malignancies, but as single agents their activity was modest and ongoing clinical trials are evaluating the role of FTIs in combination with standard cytotoxic drugs [39-42]. Our results using Ph-positive ALLs with or without the T315I mutation suggest that a combination of PHA-739358 with an FTI may be an alternative useful combination to test. Interestingly, the addition of PHA-739358 to dasatinib and vincristine, two drugs currently in clinical use, also was beneficial in terms of reducing clonogenic potential and cell killing of ALL cells. These results suggest that there may be numerous other drugs that could be combined with this Aurora kinase inhibitor, a possibility that could be rapidly evaluated in model systems such as the one used in the current study.

An international, multicenter phase I study in adult patients with advanced CML and Ph-positive ALL resistant or intolerant to imatinib or second generation of tyrosine kinase inhibitors used three cycles of PHA-739358 as a 3-hour infusion for 7 consecutive days every 2 weeks [43]. Therefore, we tested the efficacy of treatment with PHA-739358 on human Ph-positive ALL cells with the T315I mutation by administering the drug in 3 cycles of 7 days each, using a drug dose also used by Carpellini and Moll [31]. *In vivo* drug treatment was effective in ablation of the tyrosine kinase activity of the Bcr/Abl T315I mutant. While on treatment with PHA-739358, the number of circulating ALL cells was markedly suppressed and all parameters measured, including peripheral blood ALL cell counts, terminal spleen weight and overall survival show that this approach results in significant reduction of leukemia progression, but not in a cure. Based on these *in vivo* and *in vitro data*, we conclude that PHA-739358 has therapeutic effects against a variety of ALL cells, including Ph + wt, Ph + T315I and Ph- subclasses. However, increasing the dose of drug did not result in a proportional increase in cell killing and discontinuation of treatment allowed the cells to resume proliferation.

## Conclusions

We conclude that therapy with PHA-739358 may offer an alternative for patients with ALL, particularly for Ph-positive ALL patients who are intolerant to or have become resistant to imatinib, nilotinib or dasatinib with the T315I [44,45] but that combined therapy with other drugs such as a farnesyltransferase inhibitor, vincristine, or dasatinib may be needed for more effective treatment.

## Methods

### Drugs, reagents and cells

PHA-739358 (Danusertib) was provided by Nerviano Medical Sciences (Nerviano, Italy). Dasatinib was obtained commercially from Toronto Research Chemicals (Toronto, ON, Canada). PHA-739358 and dasatinib were dissolved in DMSO and stored at −80°C. The FTI SCH66336 was obtained from Schering-Plough. A vincristine sulfate solution was obtained from Hospira Worldwide Inc. (Lake Forest, IL, USA). The murine OP9 stromal cell line (CRL-2749) was obtained from the ATCC (Manassas, VA, USA). Human Ph-positive ALL cells included wild-type Bcr/Abl (UCSF02, TXL2), T315I mutants (BLQ1, Pt2) and Ph negative ALL cells (US6, US7, US7R) and were described previously [46]. US6 was from a Ph-negative ALL patient at diagnosis. The primary cells were passaged in NOD/SCIDγc−/− (NSG) mice (Jackson Labs, Bar Harbor, ME). Leukemia cells harvested from the spleens of these mice were plated on irradiated OP9 feeder layers. 8093 and Bin2 Bcr/Abl P190-expressing transgenic mouse lymphoblastic leukemia cells have been previously described [47-49] and were grown in the presence of E13.5 irradiated mouse embryonic fibroblasts (MEFs). Human leukemia cells were grown in αMEM medium supplemented with 20% FBS, 1% L-glutamine and 1% penicillin/streptomycin (Invitrogen Corporation). Mouse leukemia cells were grown in McCoy’s 5A medium including 15% FBS supplemented with 110 mg/L sodium pyruvate, 1% L-glutamine, 1% penicillin/streptomycin, 10 ng/ml recombinant IL-3 (Invitrogen Corporation, Carlsband, CA, USA) and 50 μmol/L β-mercaptoethanol.

### Analysis of cell proliferation, apoptosis and DNA content

ALL cells were cultured in a 24-well or 6-well plate at a density of 1x10^6^ cells/ml, in the presence of irradiated OP9 cells or MEFs. Cells were treated with various concentrations of PHA-739358 or SCH66336 in triplicate wells and viability of cells was measured by Trypan blue exclusion assay. Apoptotic cells were assessed by an Annexin V-fluorescein isothiocyanate (FITC) apoptosis detection kit I (BD Pharmingen, San Diego, USA). Apoptotic cells were defined by double positivity for Annexin V and PI evaluated by flow cytometry (Accuri, Ann Arbor, MI, USA). For cell cycle distribution, cells were washed and fixed in 70% ethanol for one hour. Fixed cells were stained with PI and subjected to flow cytometry.

### Assessment of phosphorylation status of histone H3 by flow cytometry

BLQ1 or US6 cells were treated with 1 μM PHA-739358 for 24 hours or 48 hours, followed by washing and fixing with 70% ethanol for one hour on ice. Cells were blocked with human FcR Blocking Reagent (Miltenyi Biotec, Bergisch Gladbach, Germany) for 10 minutes and incubated with phospho-histone H3 (Ser10) Ab (Cell Signaling Technology, Danvers, MA). After 45 minutes of incubation, cells were washed and incubated with anti-rabbit IgG FITC-conjugated antibody (Jackson ImmunoResearch, West Grove, PA, USA) for 30 minutes. Cells were washed and stained with PI before measuring by flow cytometry.

### Western blotting

BLQ1 and UCSFO2 ALL cells were treated with PHA-739358 with or without 100 nM dasatinib for 24 hours and lysed in RIPA buffer (50 mM Tris–HCl, pH 8.0, 150 mM NaCl, 1% Triton X-100, 0.5% deoxycholate, 0.1% SDS, 5 mM EDTA) containing PMSF, aprotinin, leupeptin, pepstatin A, Na-Fluoride and Na-Orthovanadate for 30 minutes on ice. Cell extracts were subjected to 8-15% sodium dodecyl sulfate-polyacrylamide gel electrophoresis (SDS-PAGE). Membranes were reacted with the following antibodies: pY-20-Horseradish peroxidase conjugated (BD-Transduction Laboratories, San Jose, CA), phospho-Src family (Tyr416), Src, phospho-Crkl, phospho-histone H3 and histone H3 (all from Cell Signaling Technology, USA), Bcr (N-20), Crkl (both from Santa Cruz Biotechnology, USA) and Gapdh (Chemicon International, USA) antibodies using standard procedures.

### Evaluation of PHA-739358 *in vivo*

All animal experiments were carried out in concordance with institutional IACUC and NIH guidelines. To evaluate the efficacy of PHA-739358 against Ph^+^ ALL with the T315I mutation *in vivo*, 2x10^6^ Pt2 cells were injected into female NSG mice (6 weeks old). Transplanted mice were treated with vehicle solution or PHA-739358 (30 mg/kg, iv, qd, 7 days on and 7 days off x 3 cycles) 7 days after transplantation. Peripheral blood was collected every two weeks after starting treatment and the percentage of leukemia cells was determined by measuring CD10^+^CD19^+^ double positive cells by flow cytometry. To further assess the immediate effect of PHA-739358 *in vivo*, mice that had developed leukemia were injected with PHA-739358 (45 mg/kg). Two hours after injection, spleen and bone marrow cells were collected and the phosphorylation status of histone H3 and Crkl, as well as total phosphotyrosine, were measured by Western blot.

### Colony formation assay

Pt2 or UCSF02 cells (2x10^4^/well) were plated in complete methylcellulose media supplemented with cytokines (MethoCult H4435, Stem Cell Technologies, Vancouver, Canada) and treated with different concentrations of PHA-739358 with or without the FTI SCH66336/Lonafarnib, vincristine or dasatinib, as indicated, in triplicate wells. Colonies consisting of > 40 cells were counted using an inverted microscope at day 10–14.

### Statistical analysis

Statistical analysis was performed with SPSS software. Data were presented as mean ± SD. Statistical significance of differences between groups was evaluated using one-way-ANOVA (post hoc Scheffe test) or paired t-test. The value of P < 0.05 was considered to be statistically significant.

## Abbreviations

CML: chronic myeloid leukemia; FITC: fluorescein isothiocyanate; FTI: farnesyltransferase inhibitor; MEFs: mouse embryonic fibroblasts; MST: median survival time; NSG: NOD/SCIDγc−/−; Ph + ALL: Philadelphia chromosome-positive acute lymphoblastic leukemia; SDS-PAGE: sodium dodecyl sulfate-polyacrylamide gel electrophoresis.

## Competing interests

JM is employed by Nerviano Medical Sciences. The other authors declare no conflict of interest.

## Author’s contributions

FF designed and performed research, collected, analyzed and interpreted data and wrote the manuscript. ML performed research and collected data; SS helped with design of experiments and performed research; JM interpreted data; JG interpreted data; NH designed experiments, analyzed and interpreted data and wrote the manuscript. All authors read and approved the final manuscript.

## Supplementary Material

Additional file 1 **Figure S1. **Dasatinib and vincristine potentiate the inhibitory effects of PHA-739358 on Ph-positive ALL cells. (A). Pt2 cells were treated with vincristine and PHA-739358 alone or together for 3 days. (B). UCSF02 cells were treated with dasatinib individually or combined with PHA-739358 for 3 days. Cell viability and viable cell numbers were assessed by Trypan blue exclusion assay. (*p< 0.05, **p<0.001). Click here for file

Additional file 2 **Figure S2. **PHA-739358 suppresses growth of Ph-positive ALL cells. Pt2 and UCSF02 cells were treated with PHA-739358 individually or combined with the FTI Lonafarnib, vincristine or dasatinib as indicated and analyzed in the colony formation assay. (*p< 0.05, **p <0.001). Click here for file
