# Supplementary figures and images for: Treatment of human pre-B acute lymphoblastic leukemia with the Aurora kinase inhibitor PHA-739358 (Danusertib)
Source: Mol Cancer. 2012 Jun 21;11:42. doi: 10.1186/1476-4598-11-42 (PMC3489684; doi:10.1186/1476-4598-11-42)

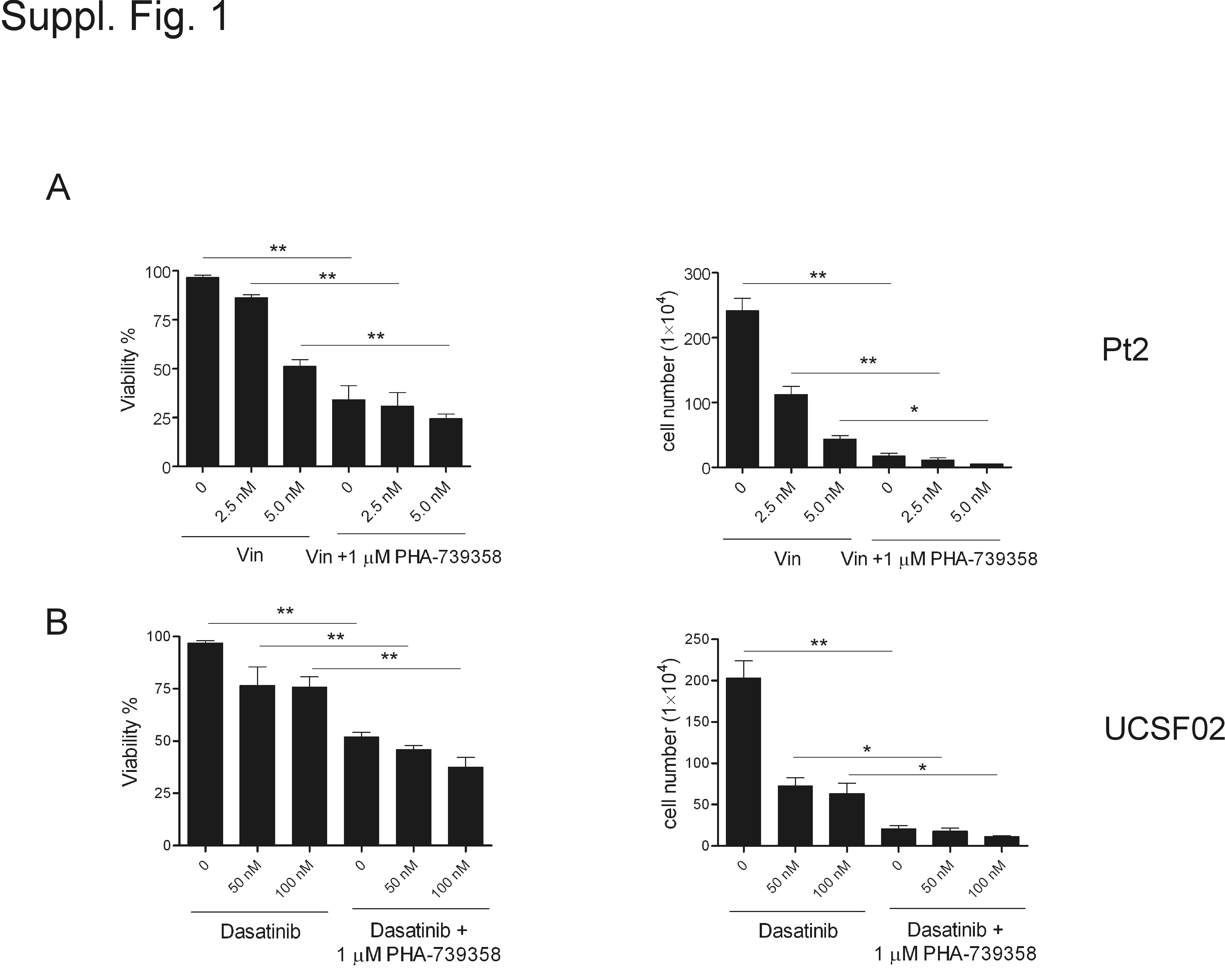

Supplement: Additional file 1 — Figure S1. Dasatinib and vincristine potentiate the inhibitory effects of PHA-739358 on Ph-positive ALL cells. (A). Pt2 cells were treated with vincristine and PHA-739358 alone or together for 3 days. (B). UCSF02 cells were treated with dasatinib individually or combined with PHA-739358 for 3 days. Cell viability and viable cell numbers were assessed by Trypan blue exclusion assay. (*p< 0.05, **p<0.001). [file 1476-4598-11-42-S1.tiff]

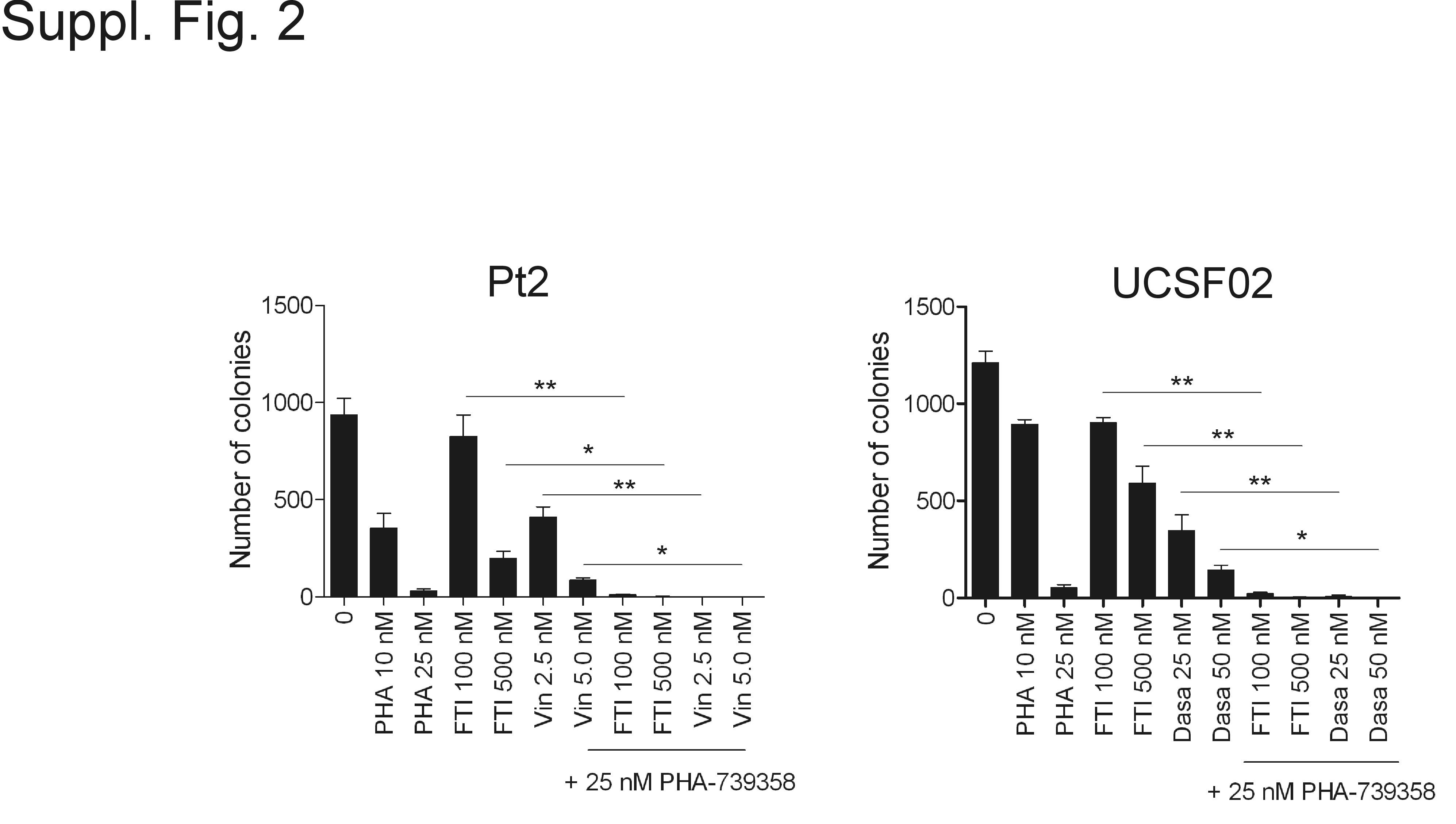

Supplement: Additional file 2 — Figure S2. PHA-739358 suppresses growth of Ph-positive ALL cells. Pt2 and UCSF02 cells were treated with PHA-739358 individually or combined with the FTI Lonafarnib, vincristine or dasatinib as indicated and analyzed in the colony formation assay. (*p< 0.05, **p <0.001). [file 1476-4598-11-42-S2.tiff]
